# Assessing the Impact of Oral Care Micro-courses on ICU Nurses’ Compliance Through a Mixed-Effects Model: A Quasi-experimental Study

**DOI:** 10.7759/cureus.75310

**Published:** 2024-12-08

**Authors:** SuWen Li, Fengzhen Wang

**Affiliations:** 1 Department of Nursing, Gannan Medical University, Ganzhou, CHN; 2 Department of Critical Care, Gannan Medical University, Ganzhou, CHN

**Keywords:** attitude, intensive care unit, mixed effects models, nurse, oral care

## Abstract

Background

Ventilator-associated pneumonia (VAP) is a common and severe hospital-acquired infection, and oral care is an effective preventive measure. However, the compliance and quality of oral care among intensive care unit (ICU) nurses need improvement.

Methods

This quasi-experimental study was conducted in two ICUs at the first affiliated hospital of Gannan Medical University, Ganzhou, China, involving 74 ICU nurses. The participants were allocated to either a micro-course education group or a conventional education group. Of these, 32 nurses were from the General ICU, and 42 were from the Cardiac ICU. Both groups received oral care education, with the micro-course group receiving video-based instruction, and the conventional group receiving PowerPoint-based training (Microsoft® Corp., Redmond, WA, USA). Data on oral care compliance and levels of Knowledge, Attitudes, and Practices (KAP) were collected at baseline and during follow-ups over one month. Statistical analysis was conducted using a mixed-effects model to compare outcomes between the groups, highlighting variations in ICU nurses' oral care practices across different ICU settings.

Results

Both education methods had statistically significant effects. The micro-course education group showed earlier and more pronounced improvements in oral care compliance. Quantitatively, the micro-course group experienced a mean increase in compliance of 0.281 (p = 0.032) at the third follow-up, whereas the conventional group saw a mean increase of 0.261 (p = 0.042) at the fifth follow-up. Additionally, KAP levels in both groups improved significantly (p < 0.001).

Conclusion

This study demonstrates that micro-course education has a statistically significant impact on ICU nurses' oral care compliance and levels of KAP in the short term. Although there was no significant difference in oral care compliance between the micro-course and conventional education methods, the micro-course showed certain advantages in teaching quality. Long-term studies are needed to evaluate the sustainability of these improvements. Promoting micro-course education in ICU nursing practice may enhance oral care practices and potentially reduce the incidence of VAP.

## Introduction

Ventilator-associated pneumonia (VAP) is the most prevalent device-related hospital-acquired infection in intensive care units (ICUs) [1]. Defined as pneumonia developing 48 to 72 hours or more after endotracheal intubation, its diagnosis requires meeting ventilator-associated conditions or infection-related ventilator-associated complications criteria, along with positive respiratory tract cultures or histopathological evidence [2]. Insufficient oral care in ICU settings contributes significantly to VAP risk, as dry oral mucosa, discomfort, pain, immunocompromised conditions, and colonisation by toxic pathogens exacerbate the problem. Between 2013 and 2015, global data from the International Nosocomial Infection Control Consortium indicated a VAP incidence rate of 9.2 cases per 1,000 ventilator days across 45 countries [3]. This critical issue prolongs ventilation duration and imposes significant financial burdens on healthcare systems, as evidenced by a recent Spanish cost assessment estimating €20,965 per VAP case [4].

Oral care is a cornerstone preventive measure for reducing VAP incidence [5]. However, studies consistently report suboptimal compliance among ICU nurses, attributed to perceptions of oral care as an unpleasant and time-consuming task [6-8]. While traditional educational interventions have demonstrated immediate improvements in care quality, the effects tend to diminish over time, highlighting the need for sustainable methods of skills retention [9-11]. Existing literature identifies multiple barriers to effective continuing education for ICU nurses, including time constraints, work commitments, limited access to training, and the perceived burden of lengthy courses [12]. Preferences for shorter, more frequent educational sessions have been documented, alongside the rising popularity of internet-based education [12,13]. These findings underscore the necessity for innovative, accessible, and engaging educational methods, tailored to the demanding ICU environment.

Micro-courses, guided by cognitive load theory, represent a novel approach to online education, offering concise, targeted teaching videos that focus on specific knowledge. By leveraging the primacy and recency effects, this format supports the transfer of information from short-term to long-term memory while minimising mental fatigue [14]. Despite the promise of micro-courses in improving learning outcomes, their application in clinical education remains underexplored, particularly in the context of ICU oral care and VAP prevention. Moreover, previous studies have rarely examined dynamic changes in compliance following multiple educational interventions, nor have they employed robust statistical approaches to account for individual variability in response to these interventions.

To address these gaps, this study integrates a mixed-effects model to evaluate compliance trajectories under repeated educational interventions, providing a nuanced analysis of factors influencing ICU nurses' adherence to oral care practices over time. Additionally, it assesses changes in Knowledge, Attitudes, and Practices (KAP) levels before and after the interventions. The study also seeks to explore the broader implications of its findings for improving ICU practices on regional and global scales by offering a scalable and effective educational framework. This framework has the potential to be widely adopted in diverse ICU settings, ultimately enhancing patient care outcomes and mitigating the risk of VAP.

## Materials and methods

Type of study and setting

This study adopts a quasi-experimental pre-test and post-test intervention design, structured as a pre- and post-control trial. The research will be conducted in the Cardiac ICU (CICU) and General ICU (GICU) of the first affiliated hospital of Gannan Medical University, Ganzhou, China, from August to December 2023. The inclusion criteria are ICU nurses who hold a valid registration certificate issued by the Chinese Ministry of Health, can understand the questionnaire content, and have voluntarily provided informed consent. Nurses currently taking psychiatric medications, or not actively employed due to marriage, illness, childbirth, or other reasons, will be excluded.

Sample size and sampling

The primary outcome of this study is the comparison of oral care practice accuracy rates one month before and one month after the intervention, analysed using the Chi-square test. Sample size estimation was based on effect sizes reported in similar studies [15-18], with an average effect size of 0.5, indicating a moderate effect of oral care education on ICU nurses' oral care compliance. The significance level was set at α = 0.05 (two-sided test), with a statistical power (1-β) of 0.80. Power analysis was conducted using PASS 2021 software, which determined that 64 nurses were required for adequate statistical power. Considering an anticipated attrition rate of 10%, a total of 70 nurse participants will be needed.

Data collection

Data collection was conducted from August to December 2023. General information was collected through questionnaires. ICU nurses' oral care compliance was assessed by trained researchers using standard protocols, while their KAP related to VAP prevention was also evaluated via validated questionnaires. Data were entered into Excel spreadsheets (Microsoft® Corp., Redmond, WA, USA) for dual verification. Consistency was ensured by conducting data collection during morning shifts (8:00-17:00) by the same researcher, adhering to strict protocols for evaluating oral care practices. Incomplete oral care indicators were excluded. Paper questionnaires were distributed and collected in person. The study indicators included: (1) oral care compliance rates, collected weekly from one month before the study, through weekly Follow-Ups II-V during the first month, and in the final week, two months after the intervention (Follow-Up VI); (2) KAP scores related to VAP prevention, assessed before and one month after the intervention, with questionnaires distributed and collected during nurse meetings.

Research tools

Oral Care Compliance Rate

The oral care compliance rate, as defined in similar studies, refers to the ratio of observed compliant oral care actions to the total oral care procedures performed. An ICU Oral Care Compliance Record was developed to measure compliance with the following criteria: (1) brushing teeth and tongue using a toothbrush for one to two minutes every 12 hours; (2) cleaning the oral and pharyngeal cavity every two to four hours; (3) using prescribed chlorhexidine (CHX) mouthwash or gel; (4) washing hands (or wearing gloves) before performing oral care; (5) elevating the head of the bed to 30°; (6) ensuring endotracheal tube (ETT) cuff inflation to 25-30 mmHg before oral care; (7) conducting an oral health assessment (using a standardised protocol or any approved method).

KAP Scores of ICU Nurses on VAP Prevention Oral Care

A validated Likert scale was employed to evaluate ICU nurses' KAP regarding oral care for VAP prevention. The scale consisted of 29 items across three domains: Knowledge (10 items, maximum score 23), Attitude (7 items, maximum score 35), and Practice (12 items, maximum score 60), with a total score of 118 (Supplementary Material 1). The survey, titled "ICU Nurses' Knowledge, Attitude, and Practice for VAP Prevention Oral Care," demonstrated strong internal consistency, with Cronbach’s alpha values of 0.82 (Knowledge), 0.86 (Attitude), 0.85 (Practice), and 0.834 overall. The Content Validity Index (CVI) ranged from 0.90 to 1.00 for individual items (I-CVI) and averaged 0.93 for the overall scale (S-CVI).

A pilot test conducted among 30 ICU nurses produced a Cronbach’s alpha of 0.94, with each domain scoring above 0.93. Content validity, reviewed by five critical care experts, yielded I-CVI values ranging from 0.80 to 1.00, and an S-CVI of 0.88 (Supplementary Material 1).

Intervention

The micro-course content includes the following modules: (1) introduction to VAP; (2) oral care assessment; (3) oral care for adult patients with orotracheal intubation under mechanical ventilation - standards by the Chinese Nursing Association; (4) explanation of the British Association of Critical Care Nurses (BACCN) oral care consensus for ICU patients [19]; (5) management of procedural pain during oral care for ICU patients with tracheal intubation and mechanical ventilation.

The micro-course was developed by the researcher under the guidance of experienced instructors in the research group. The intervention frequency was based on the Ebbinghaus forgetting curve [20], which demonstrates that the rate of forgetting is highest on the first day and then slows until reaching a relatively stable level. Accordingly, participants were instructed to study on Days 1, 4, 7, 15, and 30 of the intervention, aligning with the fourth to eighth memory cycles of the Ebbinghaus review method to facilitate memory retention and consolidation.

The experimental group intervention was conducted in the CICU demonstration room, where the researcher played the micro-course videos. Participants attended group learning sessions during morning shift handover (8:00-8:30), with each session lasting 20 minutes. After the initial session, the micro-course videos were distributed for individual review. If a participant missed a scheduled group session, a makeup session lasting 20 minutes was conducted the following day in the demonstration room. Failure to complete the makeup session resulted in exclusion from the study.

The conventional group received education with the same content as the micro-course. However, the teaching method consisted of centralised lectures using PowerPoint presentations (Microsoft® Corp., Redmond, WA, USA). Participants in the control group attended group learning sessions in their respective ICUs, each lasting 20 minutes. After the initial session, PowerPoint slides were distributed for individual review. If a participant missed a group session, a 20-minute makeup session was conducted the following day in the demonstration room. Failure to attend the makeup session resulted in exclusion from the study.

Statistical analysis

Data analysis was performed using RStudio 4.2.3 software (R Foundation for Statistical Computing, Vienna, Austria) [21]. For repeated measures of oral care compliance, a linear mixed-effects model was used to analyse time effects and random effects [22]. Results reported included estimates, standard errors, confidence intervals, and p-values, with significance set at p < 0.05. Paired t-tests or Wilcoxon signed-rank tests were used for KAP scores, depending on data normality, with a two-tailed p-value < 0.05 considered statistically significant.

Ethical considerations

This study complied with the ethical standards of the responsible institution on human subjects, as well as the Helsinki Declaration. It was approved by the Medical Ethics Committee of the First Affiliated Hospital of Gannan Medical University (LLSC-2023-NO.473). All participants provided informed consent and voluntarily and anonymously participated, with no adverse consequences for non-participation. To protect privacy, only basic information was collected anonymously, without recording identifiable personal data. All researchers agreed to maintain confidentiality, and the data were used solely for research purposes.

## Results

**Figure 1:**
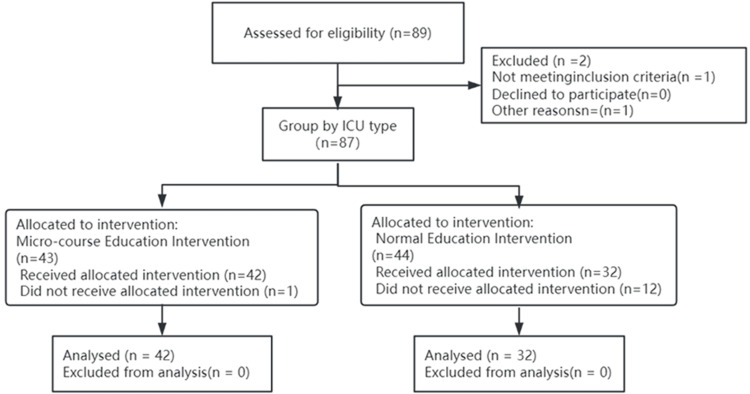
CONSORT 2010 flow diagram

A total of 87 ICU nurses from two participating ICUs were initially enrolled in the study. During the intervention, 12 participants dropped out, leaving 74 ICU nurses included in the final analysis (Figure 1). Baseline characteristics showed no significant differences in age and years of ICU experience between the nurses (Table 1). This serves as a prerequisite for ensuring the internal validity of the study.

**Table 1:** Baseline characteristics of participants

	General ICU (n = 32)	Cardiac ICU (n = 42)	p (independent-samples t-test)
Age	26.75 ± 2.13	26.25 ± 2.18	p > 0.05
Years of working in ICU	4.85 ± 2.34	5.2 ± 2.36	p > 0.05

**Figure 2:**
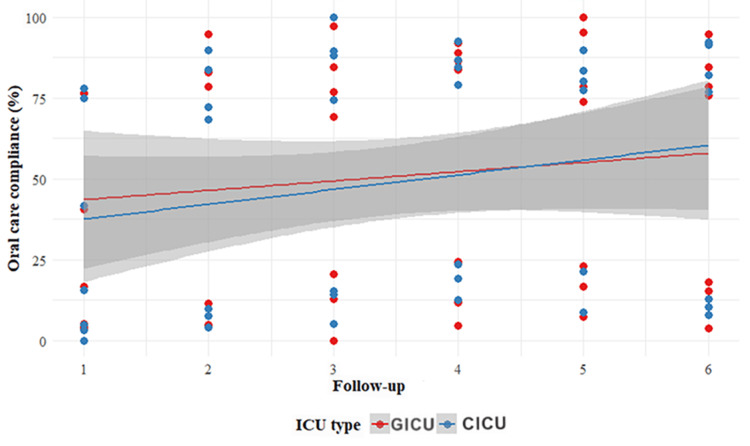
Trends in oral care indicators X-axis (Follow-Up) Description: The X-axis represents follow-up time points (1-6) in the study, assessing oral care compliance in the General ICU (GICU) and Cardiac ICU (CICU). Specific Time Points (1-6): Follow-Up I (baseline): One-month pre-intervention, establishing initial compliance levels for comparison; Follow-Ups II-V: Weekly assessments from one to four weeks post-intervention, tracking short-term compliance changes and trends; Follow-Up VI: Conducted two months after the start of the intervention, this follow-up serves as a key point for a one-week data collection period to evaluate the long-term sustainability of compliance improvements. The Y-axis represents oral care compliance as a percentage (%). The red dots and line correspond to data from the General ICU (GICU), while the blue dots and line represent data from the Cardiac ICU (CICU). The grey shaded area indicates the 95% confidence interval, highlighting the variability in the data. The red and blue lines are fitted linear regression lines for the GICU and CICU, respectively, illustrating the trends in oral care compliance over the follow-up period.

In this study, we conducted a detailed observation and analysis of oral care compliance among nurses in both the GICU and CICU. Overall, a positive trend in compliance was observed over time in both ICUs (Figure 2). However, a slight decline in the use of CHX prescriptions was noted in the CICU (Table 2). This could be attributed to various factors, such as individual patient differences, the urgency of clinical tasks, or fluctuating emphasis on CHX use during the intervention period.

**Table 2:** Oral care compliance (number of compliant observations vs. total observations) GI refers to the General ICU (control group), and CI denotes the Cardiac ICU (experimental group). Data are presented as fractions (e.g., "98/241"), where the numerator indicates compliant observations for a specific oral care procedure, and the denominator represents the total observations. These fractions calculate compliance percentages to assess oral care implementation.

Time/item	Brushing teeth once every 12 hours, using a toothbrush to clean teeth and tongue for 1-2 minutes (%)		Oral and pharyngeal cleaning every 2-4 hours (%)		Prescription use of chlorhexidine oral rinse or gel (%)		Handwashing (or glove use) before procedures (%)		Elevating the head of the bed by 30° (%)		Confirming endotracheal tube cuff inflation at 25-30 before oral care (%)		Oral health assessment (%)	
ICU type	GI	CI	GI	CI	GI	CI	GI	CI	GI	CI	GI	CI	GI	CI
Baseline period	98/241	107/257	2/122	3/125	40/241	40/257	177/231	198/264	184/241	197/237	0/152	0/184	7/137	5/153
Follow-Up II	29/37	28/41	1/23	2/25	35/37	36/39	30/36	31/43	29/41	37/43	2/40	4/41	4/35	3/39
Follow-Up III	27/39	37/42	0/17	1/19	35/35	32/32	33/39	42/47	30/39	32/47	5/39	7/46	8/39	6/42
Follow-Up IV	32/37	39/45	1/22	2/16	37/37	27/27	30/36	30/38	33/37	33/45	9/37	8/42	10/34	9/38
Follow-Up V	31/42	32/40	2/27	2/23	42/42	29/29	35/42	33/40	40/42	34/42	7/42	6/42	6/39	9/42
Follow-Up VI	28/37	30/39	1/26	2/25	37/37	35/35	31/37	32/39	31/39	36/39	7/39	7/39	7/39	5/39

At baseline (Follow-Up I), the compliance rates for various oral care practices were generally low and uneven in both the GICU and CICU. In the GICU, compliance with toothbrushing and tongue cleaning was approximately 41.6%, while in the CICU, it was about 39.8%. However, compliance with confirming ETT cuff inflation was 0% in both ICUs.

During the follow-up period, overall compliance showed an upward trend in both units. By the third follow-up (Follow-Up III), the GICU's compliance with toothbrushing and tongue cleaning increased to approximately 88.1%, with compliance for oral and pharyngeal cleaning reaching 5.3%, and ETT cuff inflation confirmation rising to 12.8%. In the CICU, compliance with toothbrushing and tongue cleaning was around 69.2%, while ETT cuff inflation confirmation improved to 20.5%.

By the sixth follow-up (Follow-Up VI), compliance levels in the GICU remained high, with toothbrushing and tongue cleaning at 73.8%, oral and pharyngeal cleaning at 76.9%, and ETT cuff inflation confirmation at 17.9%. Similarly, the CICU achieved compliance rates of 76.2% for toothbrushing and tongue cleaning, 75.7% for oral and pharyngeal cleaning, and 17.5% for ETT cuff inflation confirmation. Notably, the CICU showed higher compliance in toothbrushing and tongue cleaning compared to the GICU.

Among oral care practices, brushing teeth every 12 hours showed notably high compliance in both ICUs. By the fifth follow-up, the compliance rate in the GICU reached 73.8%, while the CICU achieved an even higher rate of 76.2%. This may be due to the relative simplicity of the procedure and the nurses' strong awareness of its importance.

In contrast, compliance with pre-oral care confirmation of endotracheal cuff inflation was initially negligible, with a baseline of 0% in both ICUs. However, by the sixth follow-up, this rate had increased to 17.9% in the GICU and 17.5% in the CICU. This indicates that, while this practice was initially overlooked, the intervention helped raise awareness, leading to gradual improvements in compliance.

Oral health assessments also showed increased compliance, with rates in the GICU improving from 5.1% at baseline to 17.9% at the sixth follow-up, and in the CICU, from 3.3% to 11.9%. These findings suggest growing attention to oral health evaluations among nurses, though there remains significant room for improvement.

By the sixth follow-up, compliance rates for other oral care practices in the GICU ranged from 40.5% to 95.2%, while those in the CICU ranged from 41.7% to 100%. These variations highlight differences in compliance based on the complexity of procedures, their direct impact on patient outcomes, and the focus of nurse training. Figure 2 illustrates these trends clearly, with the X-axis representing follow-up time points (1-6) and providing a timeline from baseline (Follow-Up I) through subsequent weekly evaluations (Follow-Ups II-V) to a key assessment two months post-intervention (Follow-Up VI). The Y-axis represents the percentage of compliance, with red and blue points and lines denoting data for the GICU and CICU, respectively. The shaded grey areas indicate the 95% confidence intervals, offering insights into data variability and reliability. Two fitted linear regression lines further emphasise the overall trend of improved compliance over time in both ICUs.

Statistical results (Table 3) confirm that both intervention methods significantly enhanced oral care compliance among ICU nurses. The Chi-square test results in Table 3 provide quantitative evidence of the significant improvement. By comparing the observed and expected frequencies of compliant and non-compliant behaviours before and after the interventions, the calculated Chi-square values and corresponding low p-values (less than the significance level, e.g., 0.05) clearly demonstrate that the changes in oral care compliance are not due to chance, but are indeed a result of the implemented interventions. This statistical analysis gives strong support to the conclusion that both micro-course education and conventional education had a meaningful impact on enhancing the oral care practices of ICU nurses.

**Table 3:** Chi-square test of the accuracy rate of ICU oral care indicators before and after intervention

ICU type	X^2^	p-value (before vs. after)
General ICU	97.81	p < 0.001
Cardiac ICU	87.31	p < 0.001

From the statistical analysis, we employed a linear mixed-effects model to assess the oral care compliance data (Table 4). This model accounts for both time effects and random effects, allowing for a more accurate evaluation of the impact of the intervention on oral care compliance. The time effect was found to be significant (p < 0.05), indicating a notable change in compliance over time, which aligns with the trend shown by the regression lines.

**Table 4:** Difference analysis of oral care compliance between conventional education and micro-course education p-values are obtained through the Wald test.

	Conventional education		Micro-course education	
	β	p-value	β	p-value
Baseline period vs. Follow-Up II	0.239	0.055	0.189	0.147
Baseline period vs. Follow-Up III	0.204	0.146	0.281	0.032
Baseline period vs. Follow-Up IV	0.243	0.065	0.226	0.091
Baseline period vs. Follow-Up V	0.261	0.042	0.201	0.139
Baseline period vs. Follow-Up VI	0.205	0.112	0.227	0.073
Follow-Up II vs. Follow-Up III	-0.001	0.982	0.033	0.352
Follow-Up II vs. Follow-Up IV	0.047	0.223	-0.009	0.830
Follow-Up II vs. Follow-Up V	0.065	0.075	-0.033	0.401
Follow-Up II vs. Follow-Up VI	0.020	0.513	0.004	0.901
Follow-Up III vs. Follow-Up IV	-0.007	0.851	0.036	0.289
Follow-Up III vs. Follow-Up V	0.011	0.769	0.022	0.539
Follow-Up III vs. Follow-Up VI	-0.036	0.233	0.055	0.055
Follow-Up IV vs. Follow-Up V	0.044	0.090	-0.028	0.300
Follow-Up IV vs. Follow-Up VI	0.008	0.772	0.012	0.663
Follow-Up V vs. Follow-Up VI	0.025	0.352	-0.007	0.810

Regarding the random effects, the analysis of variance and standard deviations at different follow-up stages (e.g., variance of 1131.36 and standard deviation of 33.63 at baseline) revealed the degree of variation in the oral care compliance indicators at both baseline and subsequent follow-up points (Table 5). The larger variance and standard deviations at baseline suggest that there was considerable variability in the performance of different oral care practices at the initial stage. In contrast, the smaller residual variance (19.59) and standard deviation (4.43) indicate a good model fit, with minimal impact from residuals, further reinforcing the reliability of the statistical results.

**Table 5:** Variability of compliance with different oral care indicators

Follow-up stage	Variance	Standard deviation	Correlation coefficient 1	Correlation coefficient 2	Correlation coefficient 3	Correlation coefficient 4	Correlation coefficient 5
Initial level of compliance variability	1131.36	33.63	/	/	/	/	/
Follow-Up II of compliance variability	776.1	27.86	-0.19	/	/	/	/
Follow-Up III of compliance variability	861.1	29.34	-0.19	1	/	/	/
Follow-Up IV of compliance variability	687.56	26.22	-0.21	0.99	0.99	/	/
Follow-Up V of compliance variability	686.6	26.2	-0.25	0.99	0.99	1	/
Follow-Up VI of compliance variability	692.96	26.32	-0.15	1	1	0.99	0.99
Residual	19.59	4.43	/	/	/	/	/

In the third follow-up (Follow-Up III), oral care compliance significantly increased in the micro-course education group compared to baseline (Follow-Up I), with an average increase of 0.281 (p = 0.032). Similarly, in the fifth follow-up (Follow-Up V), oral care compliance significantly increased in the conventional education group, with an average increase of 0.261 (p = 0.042). These findings suggest that both education methods effectively improved the oral care compliance of ICU nurses, with micro-course education demonstrating earlier and more significant effects. There were no significant differences between the two education methods at other time points (p > 0.05). Compared to baseline, all subsequent follow-ups showed improvements in compliance (Table 4), although the overall mean values were mostly non-significant. Furthermore, the overall mean values of intervention effects at the remaining follow-up time points showed no significant differences between the two education methods, indicating that both methods were influenced by time but remained effective. Micro-course education performed similarly to conventional education throughout the two-month follow-up period, maintaining relatively high compliance even after two months. This suggests that micro-course education may have a lasting impact on ICU nurses' oral care compliance, rather than just providing a temporary improvement.

From Table 5, it can be observed that the variance and standard deviation of the variability of initial levels of oral care compliance for different indicators are the largest, indicating that different indicators have a significant impact on the baseline level of oral care compliance. The variance and standard deviation of the residuals are the smallest, indicating a high model fit, with minimal impact from residuals on the results. The correlation coefficients in the model are all negative or close to zero, indicating either a negative or no linear relationship between different random effects. This suggests that the influence of different ICU types on oral care compliance is independent of each other, rather than mutually influential, demonstrating that there was no contamination in the experimental process.

Following the intervention (Table 6), there were significant improvements in the knowledge, belief, and behaviour scores in both ICUs. The Wilcoxon signed-rank test results indicate that, following the intervention, the knowledge scores in the comprehensive ICU, as well as the knowledge, belief, and behaviour scores in the CICU, were significantly higher than before the intervention (p < 0.001).

**Table 6:** Comparison of KAP before and after intervention A validated Likert scale was used to assess ICU nurses' KAP regarding oral care for VAP prevention, consisting of 29 items across three domains: Knowledge (10 items, max 23 points), Attitude (7 items, max 35 points), and Practice (12 items, max 60 points), with a total possible score of 118 (Supplementary Material 1). KAP, Knowledge, Attitude, and Practice

		Knowledge score	Attitude score	Behaviour score	Total score
Baseline	General ICU	23.9 ± 4.83	28.3 ± 5.4	35.8 ± 7.20	88 ± 17.1
	Cardiac ICU	22.55 ± 5.3	27.5 ± 5.34	34.75 ± 7.4	84.8 ± 17.75
End of follow-up	General ICU	27.7 ± 1.59	30.8 ± 2.51	40.85 ± 3.66	88 ± 17.1
	Cardiac ICU	27.65 ± 1.81	31.2 ± 2.57	41.15 ± 3.53	84.8 ± 17.75
p-value (paired-sample t-test)	General ICU	p = 0.002	p = 0.068	p = 0.007	p = 0.008
	Cardiac ICU	p＜0.001	p = 0.008	p = 0.001	p = 0.001

## Discussion

This study aimed to investigate the effects of single-dose micro-course education compared to conventional education on ICU nurses' oral care compliance and KAP levels. Baseline measurements revealed very poor oral care compliance among ICU nurses, consistent with previous literature [23]. In this study, half of the participants had less than five years of work experience, and senior title holders were rare, which may partially explain these low baseline values. Previous literature has shown correlations between ICU experience (≥7 years), senior nursing qualifications, team leadership, age, male gender, ICU type, and bachelor's degree education with higher scores [24]. This finding underscores the importance of understanding the diverse baseline behaviours of ICU nurses when designing interventions. The significant variation in compliance at baseline suggests that a one-size-fits-all approach may not be effective, and targeted, personalised education programs could help address these disparities and improve overall adherence to oral care practices.

Following the intervention, there was some improvement in oral care compliance and KAP levels. However, the effect of education methods on oral care compliance was not significant. Micro-course education significantly improved the KAP level of ICU nurses, while conventional education showed significant improvement only in knowledge scores. Comparisons with previous studies revealed both similarities and differences. Similar to Al-Abdely et al., Negm et al., and Yilmaz et al. studies, our research found that oral care education can improve ICU nurses' oral care compliance, albeit significantly evident only in Follow-Up VI [15,16,18]. However, unlike these studies, which employed complex bundles of interventions such as education, seminars, audits and feedback, and organisational changes, our study used a single-factor micro-course education - a novel and innovative oral care knowledge education method [12]. To verify its effectiveness, we compared it with conventional education, and the results showed that micro-course education was more effective in engaging and satisfying participants' learning needs and interests, similar to recent research findings [25].

To the best of our knowledge, this study may be the first attempt to investigate the dynamic changes in oral care compliance among ICU nurses using mixed-effects models. The results showed that education can effectively improve ICU nurses' oral care compliance and KAP levels, which is consistent with previous research [26]. The study also revealed that the initial level of random effect correlation coefficients and the variability between different indicators exhibited different variances. This implies that, for specific oral care indicators, such as oral and pharyngeal cleaning every two to four hours and confirming ETT cuff inflation before oral care, there were significant differences in compliance compared to other indicators. This phenomenon is also reflected in some bundle education results for preventing VAP, such as the Al-Abdely et al. study, where compliance with condensate water drainage was significantly different from compliance with other VAP prevention bundle indicators [15]. Several hypotheses were proposed to explain this phenomenon: first, ICU nurses are busy, and some operations that nurses believe can be omitted may be overlooked in clinical practice; second, although many KAP studies show that ICU nurses attach great importance to oral care, some studies report low compliance with oral care guidelines; third, due to nurses' focus on life-sustaining interventions to support critically ill patients, adhering to oral care practices is often challenging in the complex ICU environment.

Comparing the baseline with the end of the follow-up, it was observed that the compliance with oral health assessment did not increase. However, an increase in ICU nurses' attention to oral health was observed during the experiment, but they still tended to rely more on subjective judgment. This finding is consistent with some previous research [27]. Prior to data collection, strict restrictions were imposed on the standard conditions for each indicator, rather than relying on subjective judgment. Regarding oral care assessment, the 2021 British Critical Care Society pointed out that there is no requirement for a standardised ICU oral care health assessment tool [19], but it must be uniform. Due to the lack of a unified assessment tool in the experimental department, observers strictly adhered to the requirement that the observed individuals must use a written oral health assessment, such as the Beck assessment tool [28], rather than relying on subjective judgment, which may be one of the reasons for the less-than-ideal results.

We consider the dynamic impact of education on ICU nurses to be a highly significant exploration, providing new perspectives and evidence for understanding behavioural changes among ICU nurses. This study investigated the trend of oral care compliance following short-term repeated education interventions, comparing the effects of micro-course education and conventional education on compliance. At Follow-Up III, the average compliance with oral care protocols in the micro-course education group significantly increased by 0.281 compared to the baseline (p = 0.032). This result highlights the early and significant impact of micro-course education. In contrast, at Follow-Up V, the average compliance with oral care protocols in the conventional education group increased by 0.261 compared to the baseline (p = 0.042). Although both education methods led to improvements in compliance, micro-course education showed a more pronounced effect in the early phase and a faster rate of improvement.

The significance of this early effect warrants particular emphasis. The findings suggest that micro-course education not only improves ICU nurses' knowledge levels but also directly influences their behavioural changes, leading to sustained improvements in care practices. This rapid and significant enhancement of oral care compliance in the early phase supports the idea that micro-course education can stimulate nurses' attention and engagement with oral care more effectively than conventional education. This is consistent with the assumptions of behaviour change theory, which posits that early interventions can yield immediate and long-lasting effects on behaviour [29]. Moreover, micro-course education demonstrated notable advantages in teaching evaluations, particularly regarding the relevance of the content and participants' satisfaction. This further indicates that micro-course education effectively enhances ICU nurses' intention to perform oral care, thereby driving changes in their behaviour, which aligns with the theoretical assumptions of planned behaviour theory [30].

Our findings also support the assumptions of teaching evaluation theory, which suggests that teaching evaluations can reflect both the effectiveness and quality of teaching methods, as well as learners' learning outcomes. Therefore, promoting micro-course education in ICU nursing practice may not only improve nurses' KAP but also elevate the overall quality of nursing education.

Limitations

Our study, being exploratory in nature, has certain limitations. First, although oral care compliance was directly observed by trained researchers, the data on KAP levels were self-reported, with a short assessment interval of one month, potentially introducing social desirability and recall biases. Future studies should incorporate objective compliance measures, such as electronic monitoring, to mitigate these biases. Second, the small-scale design, involving only two ICUs (CICU and GICU), limits the generalisability of our findings. The differences between these ICU settings could introduce biases related to the working environment and patient population, which may affect the outcomes. Third, the restricted sample size and single-site nature constrain the external validity of the results. Future research should address this by conducting multi-centre trials with larger, more diverse samples to enhance robustness and applicability. Fourth, the study was not blinded, and observers were not rotated, which may have led to the Hawthorne effect, potentially influencing the outcomes. Incorporating blinded evaluation methods in future studies is recommended to reduce such biases. Despite these limitations, the insights gained provide valuable groundwork for more comprehensive future research.

## Conclusions

This study shows that both micro-course and conventional education effectively improve oral care compliance and KAP levels among ICU nurses. While micro-course education demonstrated some initial advantages in engagement and early impact, conventional education also led to significant improvements. Both methods are valuable in enhancing oral care practices, and future research should explore how they can be integrated or optimised for sustained improvements.

## Appendices

Supplementary material 1

ICU nurses’ oral care for VAP prevention knowledge, attitude and practice (KAP) survey

Dear nursing colleagues: Hello! This survey aims to obtain the cognitive situation of ICU oral care for preventing ventilator-associated pneumonia (VAP) and to provide a reference basis for better carrying out oral care education and training for nurses in the future. We sincerely hope that you can fill in each question truthfully and that there is no right or wrong answer for the selected answers. Do not miss any questions. The overall data analysis of this survey is only used for statistical analysis and absolutely ensures the security of your personal information.

1. The explain of “comparative agreed”:

“Comparative agreed” implies a moderate level of agreement. It indicates that the respondent has a certain degree of recognition and acceptance of the statement or situation described in the question, but not an extremely strong or absolute agreement. It falls between “agreed” and “strongly agreed” on the Likert scale. For example, when asked about the importance of oral care for preventing ventilator-associated pneumonia, if a nurse selects “comparative agreed,” it means they understand and believe that oral care is important in this context, but they may have some reservations or think that there are other factors that also need to be considered. It shows that they are in favour of the idea but not with the highest level of conviction.

2. The explain of “relatively match”:

“Relatively match” is used to describe the degree of correspondence between the respondent's actual behaviour or practice and a certain standard, expectation, or description in the question. It suggests that the respondent's actions are somewhat in line with what is being asked, but not completely or perfectly. For instance, in a question about following the department's standard oral care procedures for patients with tracheal intubation, if a nurse chooses “relatively match,” it means that they generally adhere to the procedures, but there may be occasional deviations or situations where they cannot fully comply due to various reasons such as time constraints, patient emergencies, or individual patient differences. It indicates a level of consistency that is not absolute but shows a tendency to conform to the expected behaviour.

Instructions: Please fill in the corresponding content in the blanks, and mark “√” on the appropriate numerical options.

1. Your age (single choice)*

○①20-25 age

○②26-30 age

○③31-35 age

○④36-40 age

○⑤41-45 age

2. Your gender (single choice)*

○①male

○②female

3. Your hospital grade (single choice)*

○①Grade III-A

○②Grade III-B

○③Grade II-A

4. Are you an ICU speciality nurse? (single choice)* 

○①yes

○②no

5. Years of work experience: (single choice)* 

○①1-5 years

○②6-10 years

○③11-15 years

○④16-20 years

○⑤<20 years

6. Years of ICU experience (single choice)* 

○①1-5 years

○②6-10 years

○③11-15 years

○④16-20 years

○⑤<20 years

7. Professional title (single choice)* 

○①Nurse

○②Nurse Practitioner

○③Head Nurse

○④Deputy Chief Nurse

○⑤Chief Nurse

8. Position (single choice)* 

○①Nurse

○②Nursing Team Leader

○③Deputy Director of Nursing

○④Head Nurse

○⑤Other

9. Educational background (single choice)* 

○①Vocational middle school

○②Junior College

○③Bachelor’s Degree

○④Postgraduate

10. Current marital status (single choice)* 

○①Married

○②Single

11. Employment form (single choice)* 

○①Contract-based

○②Personnel Agency

○③Permanent Staff

12. Current department is (single choice)* 

○①General ICU

○②General Surgery ICU

○③Cardiology ICU

○④Cardiothoracic ICU

○⑤Neurosurgery ICU

○⑥Emergency ICU

○⑦Respiratory ICU

○⑧Other ICU _________________

13. The number of beds in your ICU is about (single choice)* 

○①≤10

○②11-20

○③21-30

○④>30

ICU nurses’ knowledge of oral care for preventing ventilator-associated pneumonia (VAP) survey (Instructions: Please mark “√” on the appropriate number according to your understanding of the following questions)

The subjective part

14. Do you know the significance of oral care for critically ill patients with tracheal intubation? (single choice)*

○①do not know at all

○②know a little, but not exactly

○③clear

○④very clear

15. Do you know what is evaluated in oral care for critically ill patients with tracheal intubation? (single choice)*

○①do not know at all

○②know a little, but not exactly

○③clear

○④very clear

16. Do you know the choice of oral care solution for different oral PH ?(single choice)*

○①do not know at all

○②know a little, but not exactly

○③clear

○④very clear

The objective part

17. Active and effective oral nursing intervention can effectively inhibit the formation of dental plaque, reduce the number of oropharyngeal colonizing bacteria, and reduce the incidence of ventilators associated pneumonia (single choice)*

○①Yes

○②Not sure

○③No

18. The formation of dental plaque and colonizing bacteria in oropharynx are the key factors to induce ventilator-associated pneumonia (single choice)*

○①Yes

○②Not sure

○③No

19. Guidelines for the Diagnosis, Prevention, and Treatment of Ventilator-Associated Pneumonia (2018) Highly recommend the use of chlorhexidine for oral care to reduce the incidence of ventilator-associated pneumonia (single choice)*

○①Yes

○②Not sure

○③No

20. Combining oral douching with scrubbing or brushing significantly reduced the risk of ventilator-associated pneumonia, bad breath, and inflammation (single choice)*

○①Yes

○②Not sure

○③No

21. The earlier the first oral care after tracheal intubation, the better the prevention of ventilator-associated pneumonia (single choice)*

○①Yes

○②Not sure

○③No

22. AACN recommends using 0.12% chlorhexidine gluconate solution twice a day for intubated patients to reduce the risk of VAP (single choice)*

○①Yes

○②Not sure

○③No

23. AACN2021 recommends oral care every 2 to 4 hours for patients with tracheal intubation (single choice)*

○①Yes

○②Not sure

○③No

Part III: Survey of ICU nurses' attitude towards oral care to prevent ventilator-associated pneumonia (Note: Please tick "√" on the number option you think is appropriate according to your understanding of the following questions)

24. Oral care is important for the prevention of ventilator-associated pneumonia (single choice)*

○①strongly disagree

○②disagree

○③neutrality

○④comparative agreed

○⑤strongly agree

25. ICU nurses should master the relevant theoretical knowledge of oral care (single choice)*

○①strongly disagree

○②disagree

○③neutrality

○④comparative agreed

○⑤strongly agree

26. ICU nurses should master the related nursing operations of oral care (single choice)*

○①strongly disagree

○②disagree

○③neutrality

○④comparative agreed

○⑤strongly agree

27. I will actively participate in the training of oral care and prevention of ventilator-associated pneumonia (single choice)*

○①strongly disagree

○②disagree

○③neutrality

○④comparative agreed

○⑤strongly agree

28. I will take the initiative to learn the latest information on oral care to prevent ventilator-associated pneumonia (single choice)*

○①strongly disagree

○②disagree

○③neutrality

○④comparative agreed

○⑤strongly agree

29. Oral care for the prevention of ventilator-associated pneumonia should be performed by trained nurses (single choice)*

○①strongly disagree

○②disagree

○③neutrality

○④comparative agreed

○⑤strongly agree

30. The department conducted effective supervision on the implementation of oral care to prevent ventilator-associated pneumonia (single choice)*

○①strongly disagree

○②disagree

○③neutrality

○④comparative agreed

○⑤strongly agree

Part IV: Investigation of the behaviour of ICU nurses for oral care to prevent ventilator-associated pneumonia (Note: Please follow up your understanding of the following questions, and tick "√" on the number option you think is appropriate)

31. You use chlorhexidine solution (chlorhexidine/stomatamide) for oral care of mechanically ventilated patients (single choice)*

○①strongly Disagree

○②basically Disagree

○③basically agree

○④relatively match

○⑤totally agree

32. You assess the patient's oral hygiene daily (single choice)*

○①strongly disagree

○②basically disagree

○③basically agree

○④relatively match

○⑤totally agree

33. You will thoroughly evaluate the patient's teeth, gums, tongue, mucosa, lips, saliva, oral odours, etc (single choice)*

○①strongly disagree

○②basically disagree

○③basically agree

○④relatively match

○⑤totally agree

34. You will promptly respond to the superior doctor about the oral condition of critically ill patients with tracheal intubation (single choice)*

○①strongly disagree

○②basically disagree

○③basically agree

○④relatively match

○⑤totally agree

35. Oral care for patients with artificial airways was conducted according to the change of patients' condition and oral cleanliness (single choice)*

○①strongly disagree

○②basically disagree

○③basically agree

○④relatively match

○⑤totally agree

36. Balloon pressure was measured and maintained at 25-30 cm H_2_O before oral care for patients with tracheal intubation (single choice)*

○①strongly disagree

○②basically disagree

○③basically agree

○④relatively match

○⑤totally agree

37. You can always perform oral care for patients with tracheal intubation according to the department's standard oral care procedures (single choice)*

○①strongly disagree

○②basically disagree

○③basically agree

○④relatively match

○⑤totally agree

38. Oral care should be performed by at least 2 nurses each time for critically ill patients with tracheal intubation (single choice)*

○①strongly disagree

○②basically disagree

○③basically agree

○④relatively match

○⑤totally agree

39. You will increase the frequency of oral care for patients with bad oral odour (single choice)*

○①strongly disagree

○②basically disagree

○③basically agree

○④relatively match

○⑤totally agree

40. Oral PH is measured each time before oral care is administered to critically ill patients with tracheal intubation (single choice)*

○①strongly disagree

○②basically disagree

○③basically agree

○④relatively match

○⑤totally agree

41. Remove artificial airway sputum and oropharyngeal secretions before each oral care (single choice)*

○①strongly disagree

○②basically disagree

○③basically agree

○④relatively match

○⑤totally agree

42. Check the balloon pressure and tracheal intubation depth again after each oral care (single choice)*

○①strongly disagree

○②basically disagree

○③basically agree

○④relatively match

○⑤totally agree
